# Metabolic biomarkers related to cardiac dysfunction in metabolic-dysfunction-associated fatty liver disease: a cross-sectional analysis

**DOI:** 10.1038/s41387-022-00182-7

**Published:** 2022-01-18

**Authors:** Abdulrahman Ismaiel, Mihail Spinu, Carmen Socaciu, Livia Budisan, Daniel-Corneliu Leucuta, Stefan-Lucian Popa, Bogdan Augustin Chis, Ioana Berindan-Neagoe, Dan Mircea Olinic, Dan L. Dumitrascu

**Affiliations:** 1 2nd Department of Internal Medicine, “Iuliu Hatieganu” University of Medicine and Pharmacy, 400006 Cluj-Napoca, Romania; 2 Medical Clinic No. 1, “Iuliu Hatieganu” University of Medicine and Pharmacy, Cluj-Napoca, 400006 Romania; 3 Department of Biochemistry, University of Agricultural Sciences and Veterinary Medicine & BIODIATECH - Research Center on Applied Biotechnology in Diagnosis and Molecular Therapy, Cluj-Napoca, Romania; 4 Research Center for Functional Genomics, Biomedicine and Translational Medicine, “Iuliu Hațieganu” University of Medicine and Pharmacy, 400337 Cluj-Napoca, Romania; 5 Department of Medical Informatics and Biostatistics, “Iuliu Hatieganu” University of Medicine and Pharmacy, 400349 Cluj-Napoca, Romania; 6 Research Center for Advanced Medicine-Medfuture, Iuliu Hatieganu University of Medicine and Pharmacy, 23 Marinescu Street, 400337 Cluj-Napoca, Romania; 7 Department of Functional Genomics and Experimental Pathology, The Oncology Institute “Prof. Dr. Ion Chiricuta”, 400015 Cluj-Napoca, Romania; 8 Interventional Cardiology Department, Emergency Clinical Hospital, Cluj-Napoca, 400006 Romania

**Keywords:** Cardiovascular diseases, Endocrine system and metabolic diseases

## Abstract

**Introduction:**

Hepatic steatosis is associated with cardiac systolic and diastolic dysfunction. Therefore, we evaluated metabolites and their potential cardiovascular effects in metabolic-dysfunction-associated fatty liver disease (MAFLD).

**Materials and methods:**

We conducted a cross-sectional study involving 75 participants (38 MAFLD and 37 controls). Hepatic steatosis was confirmed by hepatic ultrasonography and SteatoTest^TM^. Cardiac function was assessed using echocardiography. Metabolomic analysis was conducted using ultra-high-performance liquid chromatography–mass spectrometry.

**Results:**

The median age for participants’ age was 45 (IQR 30–56.5), with gender distribution of 35 males and 40 females. MAFLD patients had lower levels of glycyl tyrosine (*p*-value < 0.001), lysophosphatidylcholine (LPC) (18:2/0:0) (*p*-value < 0.001), LPC (22:6) (*p*-value < 0.001), and ceramide (Cer) (d18:0/23:0) (*p*-value 0.003) compared to controls. MAFLD patients presented lower left ventricular ejection fraction (LVEF), E/A ratio, E/e′ ratio, and average global longitudinal strain (GLS) values, with a *p*-value of 0.047, <0.001, 0.008, and <0.001, respectively. Decreased glycyl tyrosine levels were significantly correlated with reduced LVEF, even after performing multiple linear regression with 95% CI (1.34–3.394, *p*-value < 0.001). Moreover, decreased LPC (18:2/0:0) levels remained significantly associated with E/A ratio, even after adjusting for confounding factors with 95% CI (0.008–0.258, *p*-value = 0.042).

**Conclusion:**

MAFLD patients are at risk for developing cardiac systolic and subclinical systolic dysfunctions, as well as diastolic dysfunction. Decreased glycyl tyrosine levels correlate with reduced LVEF and LPC (18:2/0:0) levels with diastolic dysfunction, even after adjusting for confounding factors, suggesting their potential to be used as metabolic biomarkers in detecting cardiovascular risk.

## Introduction

Metabolic‐dysfunction‐associated fatty liver disease (MAFLD) is defined by the presence of hepatic steatosis, in addition to one of the following three criteria including overweight/obesity, type 2 diabetes mellitus (DM) or metabolic dysfunction [1, 2]. The prevalence of MAFLD is rapidly growing worldwide, along with the parallel increase in metabolic diseases, while remaining without currently approved pharmacotherapies [3–6].

The pathogenesis of MAFLD is considered complex and multifactorial [7, 8]. Several metabolic biomarkers have also been recently studied in hepatic steatosis, including lysophosphatidylcholine (LPC) an endogenous phospholipid from the class glycerophospholipids, glycyl tyrosine a dipeptide composed of glycine and l-tyrosine joined by a peptide linkage, ceramides being considered as members of the class of compounds known as sphingolipids (SPs), and triglycerides being members of glycerolipids [9–13].

Several extrahepatic manifestations have been reported in NAFLD, being considered an independent risk factor for increased cardiovascular disease (CVD)‐related morbidity and all‐cause mortality [14, 15]. However, the current literature is scarce in data evaluating extrahepatic cardiovascular manifestations using the new criteria for MAFLD. Furthermore, whether hepatic steatosis per se is causally associated with an increased cardiovascular (CV) risk remains inconclusive [16–19]. Several methods have been described to evaluate cardiac systolic and diastolic functions, as well as subclinical systolic function.

The most evaluated parameter with overwhelming clinical utility to assess the cardiac systolic function is through measuring the left ventricular ejection fraction (LVEF) [20]. However, this measurement is associated with several limitations as it utilizes an indirect estimation of myocardial contractile function which cannot identify alterations in minor contractile function, as in addition to including factors that can be modified by several factors such as heart rate and loading conditions. Therefore, subclinical myocardial damage, a condition found to be implicated prognostically with multiple pathologies, cannot be appropriately assessed using LVEF.

Speckle-tracking echocardiography (STE), a novel non-invasive ultrasound imaging modality that simplifies the identification of early LV dysfunction in subjects with preserved LVEF, enhanced the evaluation of global and regional myocardial function regardless of cardiac translational movements and the insonation angle [21]. STE quantifies myocardial contraction as a percentage of the myocardial segment length difference over a specific period of time, while overcoming several drawbacks that are present in LVEF [20].

Furthermore, in order to evaluate the cardiac diastolic function, the evaluation of several velocities including early diastolic peak velocity (*E*), late diastolic peak velocity (*A*), early diastolic velocity (*e*′), and late diastolic velocity (*a*′), as well as several ratios such as *E*/*A*, *e*′/*a*′, and *E*/*e*′ are required [22].

Recently, several articles demonstrated that NAFLD is associated with an increased risk of diastolic dysfunction as well as subclinical systolic dysfunction, with inconclusive results regarding systolic dysfunction [23, 24]. However, these alterations were not evaluated using the newly defined criteria of MAFLD.

Therefore, we conducted to the best of our knowledge, the first cross-sectional study evaluating several metabolic biomarkers including LPC (18:2/0:0), LPC (22:6), Glycyl tyrosine, and Cer (d18:0/23:0) and their potential cardiovascular effects regarding systolic, subclinical systolic, and diastolic cardiac functions in MAFLD. Our hypothesis was that the evaluated metabolic biomarkers will help predict MAFLD, as well as non-invasively identify cardiovascular dysfunction.

## Materials and methods

### Study participants

We conducted an observational cross-sectional analysis involving subjects ≥ 18 and <65 years old. Inclusion criteria included subjects identified as MAFLD patients admitted at the Clinical Emergency County Hospital of Cluj-Napoca, Romania who fulfilled the diagnostic criteria of MAFLD [2]. We confirmed hepatic steatosis using both hepatic ultrasonography and SteatoTest^TM^ (BioPredictive), simultaneously for all included participants in order to improve our diagnosis of hepatic steatosis. Participants who did not fulfill the criteria with confirmed hepatic steatosis using both ultrasonography and SteatoTest^TM^ (BioPredictive) were excluded from the MAFLD group. Controls subjects were mainly healthy hospital staff that did not fulfill the diagnostic criteria for MAFLD. Recruitment of participants was performed between January 2020 and September 2020. We performed non-probability consecutive sampling of eligible subjects. Exclusion criteria for both groups including MAFLD patients and controls included subjects aged <18 and >65 years, presence of other secondary causes of hepatic fat accumulation, hepatitis B virus infection, malignant or benign liver tumor, any other coexistent liver disease, acute hemolytic diseases, acute inflammatory pathologies such as Ulcerative Colitis or Crohn’s Disease, deep venous thrombosis, any active malignancies, any active pulmonary exacerbations such as COPD exacerbation or asthma, systemic lupus erythematosus, acute infections (dental, urinary, pulmonary, flu, COVID-19, etc.), failure to fast for at least 12 h before blood tests, and refusal to participate in the study. This study was approved by the local ethical and research committee of the “Iuliu Hatieganu” University of Medicine and Pharmacy Cluj-Napoca (no. 486/21.11.2019) and was conducted according to the guidelines of the 1975 Helsinki Declaration, revised in 2013. A written informed consent was obtained from all participants.

### Data collection and general definitions

Height and body mass were assessed with subjects wearing light clothing and no shoes, and were rounded to the nearest 0.1 kg and 0.1 cm, respectively. The calculation of BMI was calculated according to body mass in kilograms divided by the square of the height in meters (kg/m^2^).

The definition of hypertension was considered according to the 2020 International Society of Hypertension Global Hypertension Practice Guidelines [25]. The diagnosis of diabetes and prediabetes were determined according to the American Diabetes Association recommendations – Classification and Diagnosis of Diabetes: Standards of Medical Care in Diabetes – 2021 [26]. Dyslipidemia was identified according to the National Cholesterol Education Program guidelines [27].

### MAFLD definition

The diagnosis of MAFLD was based on the presence of hepatic steatosis on hepatic ultrasonography and SteatoTest^TM^ (Biopredictive), in addition to one of the following criteria: (1) overweight or obesity as defined by a BMI ≥ 25.0 kg/m^2^; (2) established type 2 diabetes mellitus; or (3) confirmation of at least two metabolic risk alterations [2]. Metabolic risk alterations were defined as: (1) waist circumference ≥102/88 cm in Caucasian men and women; (2) blood pressure ≥130/85 mmHg or drug treatment; (3) plasma triglycerides ≥150 mg/dL or specific pharmacotherapy; (4) plasma HDL-C < 40 mg/dL for men and <50 mg/dL for women or specific pharmacotherapy; (5) prediabetes (i.e., fasting glucose levels 100 to 125 mg/dL, or HbA1c 5.7% to 6.4%); and (6) plasma high-sensitivity C-reactive protein (hs-CRP) levels >2 mg/L [2].

### Hepatic ultrasonography

Liver ultrasound assessment for hepatic steatosis was performed by an experienced physician who was blinded to the patients’ diagnosis, labs, and the aims of the study, using GE LOGIQ S7 Expert. Prior to the ultrasound assessment, participants were asked to fast for at least 8 h. During the ultrasound assessment, the liver parenchyma was evaluated both subcostally and intercostally. In order to find the best approach and avoid artifacts, subjects were evaluated in supine position and in modified slightly oblique position while placing the right arm above the head and having the right leg stretched during all respiration cycles. We used the following criteria in order to assess for hepatic steatosis: (1) ultrasonographic contrast between the parenchyma of the liver and right kidney; (2) liver brightness; (3) penetration of ultrasound deep attenuation into the hepatic deep portion and diaphragmatic impaired visualization; and (4) impaired visualization of intrahepatic vessels borders, as well as lumen narrowing [28].

### Echocardiography

All subjects underwent a comprehensive echocardiographic evaluation by a board-certified cardiologist who was blinded to the patients’ diagnosis, labs, and the aims of the study, using GE Vivid q Ultrasound Machine. The echocardiographic evaluation was performed independent of the metabolomic analysis. The echocardiographic evaluation included M-mode, 2-dimensional, conventional color and Doppler ultrasonography, as well as global longitudinal strain assessment, according to current recommendations and guidelines [22, 29–33]. A dedicated software for automated ejection fraction calculation was used, while border detection was verified and corrected for precision. Automated calculation of end-systolic volume (ESV) and end-diastolic volume (EDV) from the 4- and 2-chamber apical views, as well as LVEF was performed. Doppler-derived transmitral inflow profiles were obtained in apical 4-chamber views with a sample volume of 2 mm placed between the mitral leaflet tips in the apical 4-chamber view. The peak velocities of the early (*E*) and late (*A*) phases of the mitral inflow were measured from the mitral inflow Doppler assessment, and the *E*/*A* ratios were calculated automatically. LV myocardial velocities were obtained in the apical 4-chamber view with a sample volume being placed at the septal mitral annulus through Tissue Doppler imaging (TDI). Moreover, the peak velocities of early diastolic (*e*′) and late diastolic (*a*′) phases were obtained from the pulsed-wave TDI, and the *E*/*e*′ ratio was calculated automatically, indicating LV filling pressure. Using two-dimensional speckle-tracking echocardiography, we measured the Global Longitudinal Strain (GLS) and strain rate curves from all LV myocardial segments including 4-chamber, 2-chamber, and long-axis apical views. The average peak systolic longitudinal strain values and peak systolic strain rate were measured as global longitudinal strain (LS_SYS_) and global strain rate (SR_SYS_), respectively.

### Laboratory analysis

All blood samples were obtained by venipuncture into vacutainer tubes after 12 h of overnight fasting. Protocols for blood sampling and analysis of blood samples were followed.

### FibroMax

The separated sera were stored at 2 °C–8 °C for a maximum of 1 day, then assayed for the 10 serum biomarkers that are included in the FibroMax score. The obtained results were adjusted for age, gender, weight, and height.

Nephelometry from serum samples was used to assess α2-macroglobulin, haptoglobin, apolipoprotein A1, while spectrophotometry from serum samples was used to assess total bilirubin, gamma-glutamyltransferase (GGT), aspartate aminotransferase (AST), alanine aminotransferase (ALT), total cholesterol, and triglycerides. Moreover, plasma fasting glucose was assessed using NaF/K2 oxalate spectrophotometry. The parameters were assayed using BN ProSpec System from Siemens for nephelometry and Siemens Atellica from Siemens for spectrophotometry.

The results of the measured blood variables were entered into the BioPredictive network where the algorithms were computed. In this study, SteatoTest which is considered a measure of the steatosis grade in hepatocytes that ranges from S0–S3, was used in our study in combination with ultrasonography to confirm hepatic steatosis [34].

### Metabolomic analysis

Blood serum samples were collected into vacutainer tubes containing EDTA as anticoagulant. The blood plasma was obtained by centrifugation at 2000 rpm for 10 min and aliquots of 1 ml were frozen and stored at −80 °C until analysis. We added 0.8 ml of mixture methanol:acetonitril with a ratio of 1:1 95% to a volume of 0.2 ml of plasma to precipitate proteins. The mixture was vortexed for 20 s and stored at −20 °C for 24 h. After defreezing, the vials were centrifuged at 17,470×*g* for 10 min and the supernatant was collected, filtered through 0.2 µm PTFE filters and introduced in the vials for metabolomic analysis.

The ultra-high-performance liquid chromatography–mass spectrometry (UHPLC-MS) analysis was performed on a Bruker Daltonics MaXis Impact (Bruker GmbH, Bremen, Germany) device including a Thermo Scientific HPLC UltiMate 3000 system with a Dionex Ultimate quaternary pump delivery and ESI + -QTOF-MS detection, on C18 reverse-phase column (Acquity, UPLC C18 BEH) (5 µm, 2.1 × 75 mm) at 25 °C and a flow rate of 0.3 ml/min. The injection volume was 5 μl. The mobile phase was represented by a gradient of eluents A (water containing 0.1% formic acid) and eluent B (methanol:acetonitrile, 1:1, containing 0.1% formic acid). The gradient system consisted of 99% A (min 0), 70% A (min 1), 40% A (min 2), 20% A (min 6), and 100% B (min 9–10) followed by 5 min with 99% A. The total running time was 15 min.

The mass spectrometry (MS) parameters were set for a mass range between 50 and 1000 Da. The nebulizing gas pressure was set at 2.8 bar, the drying gas flow at 12 L/min, the drying gas temperature at 300 °C. Before each chromatographic run, a calibration with sodium formate was performed. The control of the instrument and data processing used the specific software provided by Bruker Daltonics, namely Chromeleon, TofControl 3.2, Hystar 3.2, and Data Analysis 4.2.

### Statistical analysis

Statistics regarding the metabolomic analysis were performed as follows. The preprocessed data were obtained using Data Analysis. Firstly, the individual total Ion Chromatograms (TIC) were registered and then transformed to Base Peak Chromatograms (BPC). Afterwards, the compound spectra were recorded using the FMF function (Find molecular features). The table released from the FMF matrix contained the retention time, peak areas and intensities, and signal/noise (S/N) ratio for each component together with its *m*/*z* value. Generally, the number of separated compounds ranged between 200 and 400.

In this first step of the statistical analysis, the matrix containing the m/z values and peak intensity for all samples was stored in an Excel file. In order to eliminate the small signals with S/N values under 3, a first filtration (1) was made and then a second matrix containing *m*/*z* values and peak intensities was saved and filtered in a second step eliminating the small intensities (<10,000) (2). Generally, the number of peaks that remained was 100–150. Only metabolites which were detected in more than 80% of the samples were included in the statistical analysis, using an alignment software from bioinformatica.isa.cnr.it/NEAPOLIS. The aligned matrix (3) offered also the possibility to calculate the mean of intensity values and standard deviation for each m/z value. By these conventional statistics, we compared the mean intensities and differences between the control group and the MAFLD group. The final aligned matrix was converted to a.csv file and introduced in the online software Metaboanalyst 4.0 (www.metaboanalyst.com).

Finally, using the Biomarker Analysis, the Receiver Operating Curves (ROC) were obtained and the values of areas under ROC curves (AUC) were obtained and the molecules identified were ranked according to their sensitivity/specificity.

The identification of the most relevant molecules which can be considered potential biomarkers was made using the two most relevant databases, LIPID MAPS® Lipidomics Gateway (https://www.lipidmaps.org/data/structure/LMSDSearch.php) and Human Metabolome Database (https://hmdb.ca/). After completing the metabolomic analysis, we chose 4 metabolites evaluated by Metaboanalyst 4.0 that were found to be significantly modified between MAFLD patients and controls using the obtained *m/z* values and conducted further evaluation using several cardiovascular parameters.

Moreover, statistical analysis was carried out using R software environment for statistical computing and graphics version 4.0.2 (R Foundation for Statistical Computing, Vienna, Austria). Continuous data were reported as mean (standard deviation, SD) – for normally distributed data or median (interquartile range) – for non-normally distributed data. Categorical data were reported as frequencies and percentages. The clinical characteristics of the study population according to the categorized groups for quantitative variables were compared with *t*-test for independent samples for normally distributed data, and with Wilcoxon rank-sum test, for non-normally distributed data, while for categorical data, *χ*2 test and Fisher exact test were used. Furthermore, to assess the relationship between the metabolic biomarkers and several echocardiographic parameters, we used univariate and multivariate linear regression models to control for confounding factors including age (years), gender, BMI (kg/m^2^), mean systolic blood pressure (mmHg), mean diastolic blood pressure (mmHg), diabetes, and MAFLD vs. control. For all linear models we checked the assumptions of residual normality (with a quantile-quantile plot), heteroskedasticity (with standardized residual vs. fitted values), the presence of high leverage, high residuals, or high influential points (with standardized residuals vs. hat-values vs. Cook’s distance plot), the linearity relation of continuous variables with the outcome (with component + residual plot). For multivariate models the presence of multicollinearity was assessed with variance inflation factors and correlation coefficients. The regression results were reported as model coefficients, 95% confidence interval (CI – computed with robust variance sandwich estimators in case of heteroskedasticity), and *p*-value. All performed statistical tests were two-sided, and a *p*-value < 0.05 was considered to be statistically significant.

## Results

### General characteristics

**Fig. 1:**
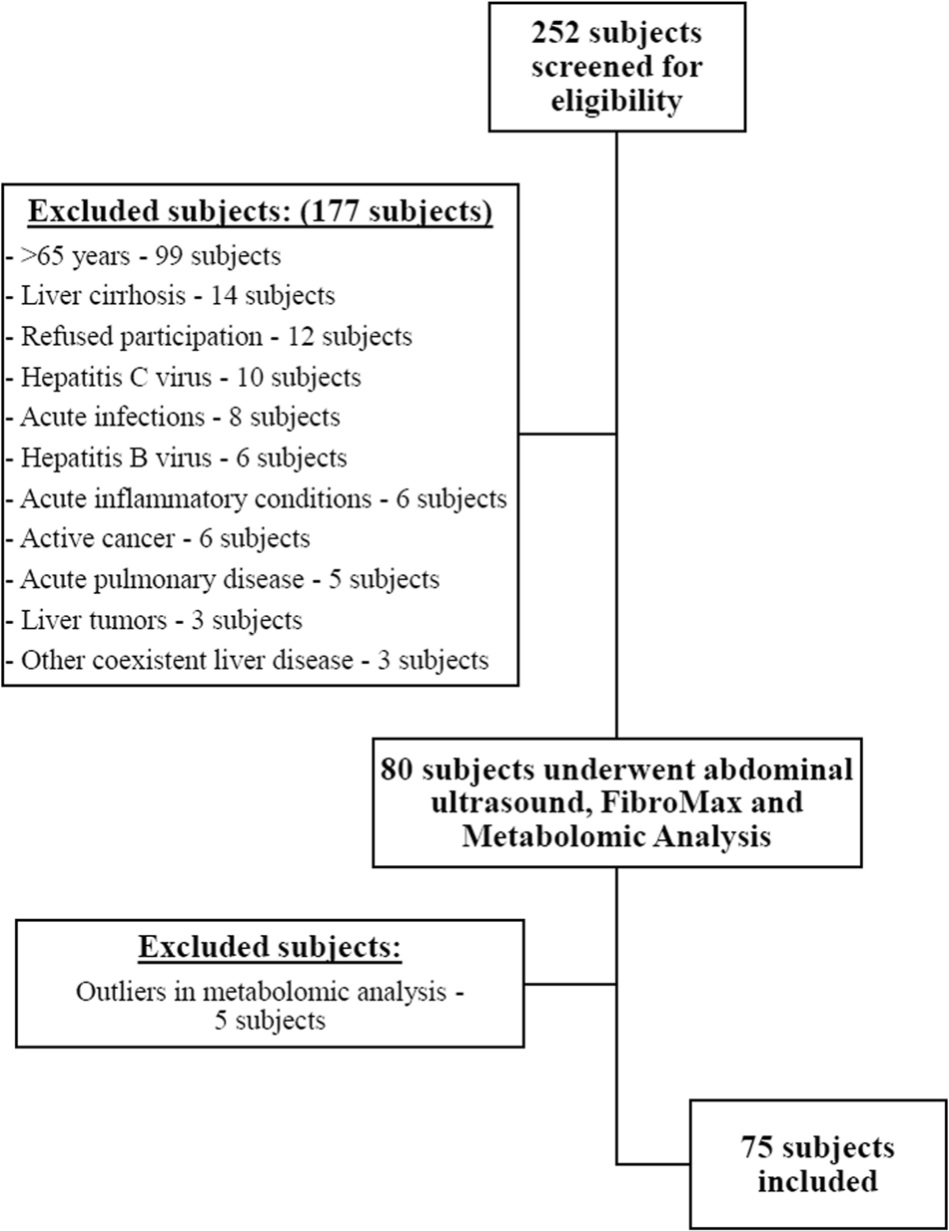
Flow diagram of included and excluded participants.

A total of 252 subjects were screened for eligibility, out of which 177 were excluded as they did not fulfill the inclusion and exclusion criteria. A flow diagram describing included and excluded participants is demonstrated in Fig. 1. After excluding ineligible subjects, a total of 75 Caucasian subjects were included in the final analysis. Table 1 summarizes the patients’ general characteristics.

**Table 1:** General characteristics of included participants.

Variable	Total (*n* = 75)	MAFLD (*n* = 38)	Controls (*n* = 37)	*P*-value
Age (years), median (IQR)	45 (30–57)	54 (48–59)	30 (27–40)	<0.001
Gender - males, no. (%)	35 (46.7)	18 (47.4)	17 (46)	0.902
BMI (kg/m^2^), median (IQR)	26.2 (22.2–30.3)	30.3 (28.1–33.3)	22.1 (20.1–24.8)	<0.001
Metabolic syndrome, no. (%)	31 (41.3)	29 (76.3)	2 (5.4)	<0.001
SBP (mmHg), median (IQR)	124 (116–134)	132 (122–147)	120 (113–124)	<0.001
DBP (mmHg), median (IQR)	78 (74–84)	82 (78–89)	75 (71–78)	<0.001
Hypertension, no. (%)	35 (46.7)	30 (79)	5 (13.5)	<0.001
Diabetes, no. (%)	16 (21.3)	16 (42.1)	0 (0)	<0.001
Impaired fasting glucose, no. (%)	5 (6.7)	3 (7.9)	2 (5.4)	1
Dyslipidemia, no. (%)	38 (50.7)	26 (68.4)	12 (32.4)	0.002
SteatoTest Score, median (IQR)	0.39 (0.13–0.635)	0.635 (0.512–0.713)	0.13 (0.08–0.19)	<0.001
SteatoTest	–	–	–	<0.001
S0, no. (%)	37 (49.3)	0 (0)	37 (100)	–
S1, no. (%)	10 (13.3)	10 (100)	0 (0)	–
S2, no. (%)	17 (22.7)	17 (100)	0 (0)	–
S3, no. (%)	11 (14.7)	11 (100)	0 (0)	–
Hepatic Steatosis (Ultrasonography), no. (%)	38 (50.7)	38 (100)	0 (0)	< 0.001

*BMI* body mass index, *DBP* diastolic blood pressure, *IQR* interquartile range, *SBP* systolic blood pressure.

Subjects were divided into two groups according to the presence or absence of MAFLD diagnostic criteria. A total of 38 subjects were diagnosed with MAFLD and 37 subjects were considered as controls. The median and IQR for the participants’ age in both groups were 45 (30–56.5), while a significant difference was found between both groups (*p*-value < 0.001). Gender distribution was divided as follows, with 35 males and 40 females, without a statistically significant difference between both groups (*p*-value = 0.902).

### Laboratory results

Compared to controls, MAFLD patients presented significantly increased BMI, systolic and diastolic blood pressures, as well as increased risk of hypertension, diabetes, dyslipidemia. All control subjects had a SteatoTest score of S0, while MAFLD patients had S1 (10 subjects), S2 (17 subjects), and S3 (11 subjects).

### Metabolomic analysis

We compared four metabolites in MAFLD patients and controls as demonstrated in Table 2. These metabolites included glycyl tyrosine, LPC (18:2/0:0), and Cer (d18:0/23:0). Interestingly, all the 3 metabolic biomarkers demonstrated a statistically significant difference between both groups incuding glycyl tyrosine (*p*-value < 0.001), LPC (18:2/0:0) (*p*-value < 0.001), LPC (22:6) (*p*-value < 0.001), and Cer (d18:0/23:0) (*p*-value = 0.003).

**Table 2:** Metabolic biomarkers in predicting MAFLD.

Metabolite	AUC	*T*-tests	Total (*n* = 75)	MAFLD (*n* = 38)	Controls (*n* = 37)	*P*-value
Glycyl tyrosine, median (IQR)	0.802	0.063	0.274 (0.212–0.325)	0.216 (0.197–0.274)	0.323 (0.272–0.358)	<0.001
LPC (18:2/0:0), median (IQR)	0.774	2.149	1.953 (0.983–3.261)	1.033 (0.364–1.597)	3.262 (2.613–4.028)	<0.001
LPC (22:6), median (IQR)	0.745	1.492	1.716 (0.972–3.061)	1.039 (0.596–1.47)	2.809 (1.669–3.432)	<0.001
Cer (d18:0/23:0), median (IQR)	0.775	1.197	1.769 (1.382–2.66)	1.323 (1.012–1.769)	2.183 (1.648–3.462)	0.003

*AUC* area under the Receiver Operating Curve, *Cer* ceramide, *IQR* interquartile range, *LPC* lysophosphatidylcholine, *MAFLD* Metabolic-dysfunction-associated fatty liver disease.

### Cardiac functional assessment

**Fig. 2:**
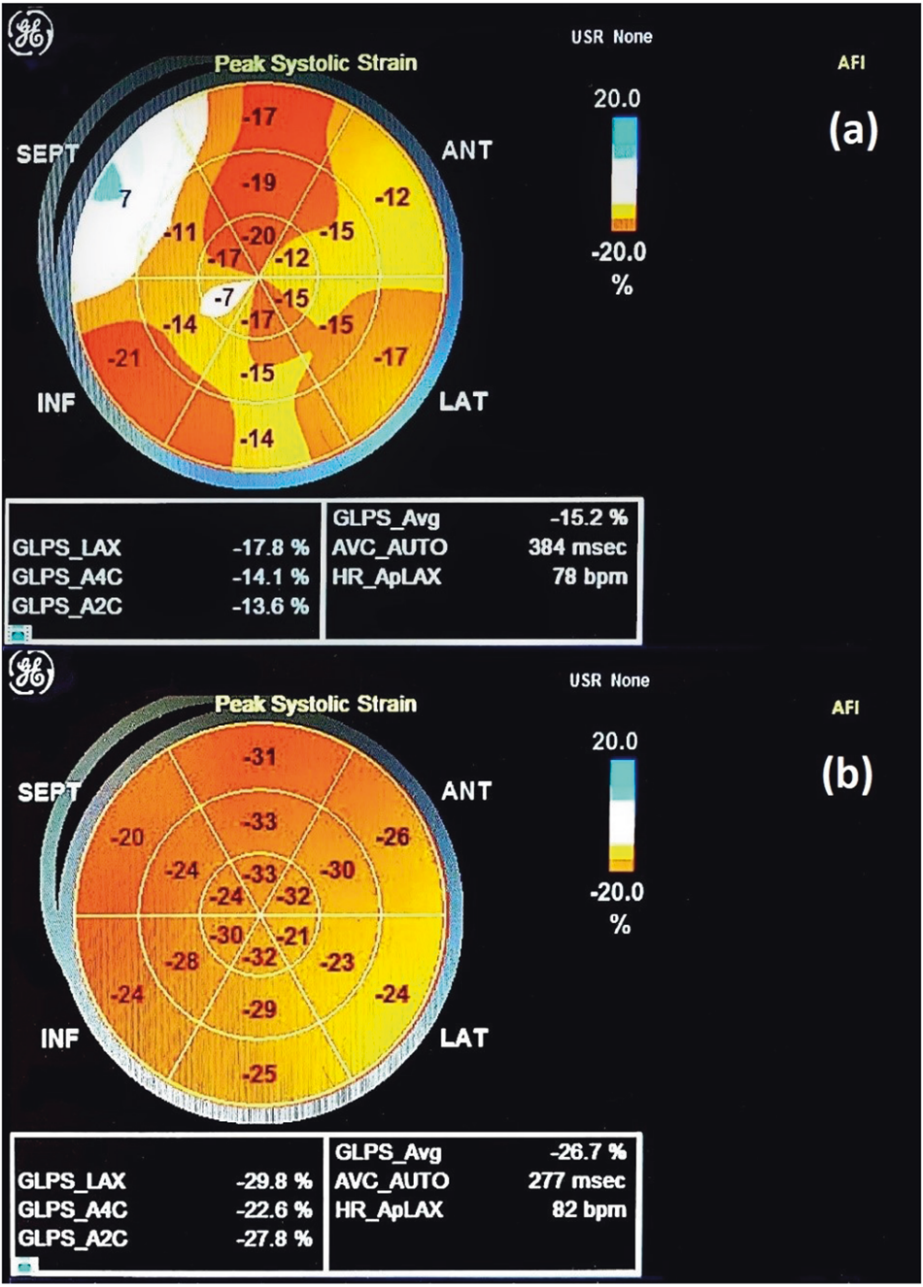
Peak systolic strain and global longitudinal strain assessment using speckle tracking echocardiography. **a** Abnormal values and **b** normal values.

We assessed several functional echocardiographic parameters as demonstrated in Table 3. The LVEF (*p*-value = 0.047), E/A ratio (*p*-value < 0.001), *e*′/*a*′ ratio (*p*-value < 0.001) and average GLS (*p*-value < 0.001) were significantly lower in MAFLD patients compared to controls. Figure 2 demonstrates peak systolic strain and global longitudinal strain findings with normal and abnormal values. On the other hand, *E*/*e*′ ratio (*p*-value = 0.008) and cardiac output (*p*-value = 0.034) were significantly increased in MAFLD patients compared to controls.

**Table 3:** Echocardiographic parameters in MAFLD patients vs. controls.

Variable	Total (*n* = 75)	MAFLD (*n* = 38)	Controls (*n* = 37)	*P*-value
LVEF (%), mean (SD)	51.187 (6.887)	49.632 (6.851)	52.784 (6.638)	0.047
Stroke volume (ml), mean (SD)	50.032 (14.104)	53.158 (13.257)	46.821 (14.4)	0.051
Cardiac output, median (IQR)	3.498 (2.815–4.35)	3.761 (3.005–5.139)	3.131 (2.574–3.96)	0.034
Early diastolic peak velocity - *E* (m/s), mean (SD)	0.76 (0.192)	0.681 (0.17)	0.842 (0.18)	<0.001
Late diastolic peak velocity - *A* (m/s), median (IQR)	0.51 (0.43–0.735)	0.72 (0.485–0.802)	0.48 (0.42–0.55)	<0.001
*E*/*A* ratio, median (IQR)	1.411 (1.007–1.819)	1.124 (0.706–1.425)	1.8 (1.364–2)	<0.001
Early diastolic velocity – *e*′ (m/s), median (IQR)	0.13 (0.105–0.17)	0.11 (0.082–0.13)	0.17 (0.14–0.2)	<0.001
Late diastolic velocity – *a*′ (m/s), median (IQR)	0.09 (0.07–0.13)	0.09 (0.08–0.158)	0.09 (0.07–0.13)	0.276
*e*′/*a*′ ratio, median (IQR)	1.5 (0.875–2.143)	1.019 (0.722–1.639)	1.7 (1.455–2.429)	<0.001
*E*/*e*′ ratio, median (IQR)	5.4 (4.393–6.833)	6.167 (4.932–7.372)	5.095 (4.048–5.7)	0.008
GLS – Long axis view (%), median (IQR)	20.1 (17.9–22.2)	18.6 (17.4–20.675)	21.4 (19.1–22.6)	0.002
GLS – 4-chamber view (%), median (IQR)	18.7 (17.1–20.6)	17.7 (15.925–19.325)	20.4 (18.2–21.9)	<0.001
GLS – 2-chamber view (%), median (IQR)	18.1 (15.5–20.7)	16.05 (14.525–17.9)	20.3 (19–21.9)	<0.001
GLS – Average (%), median (IQR)	18.967 (17.333–20.917)	17.65 (16.408–18.892)	20.733 (19.067–22.267)	<0.001

*GLS* global longitudinal strain, *IQR* interquartile range, *LVEF* left ventricular ejection fraction, *MAFLD* metabolic-dysfunction-associated fatty liver disease, *SD* standard deviation.

### Metabolic biomarkers and cardiac functional assessment

We proceeded by evaluating whether these metabolites can be considered potential metabolic biomarkers in evaluating cardiac systolic, subclinical systolic, and diastolic functions. We conducted several univariate and multivariate linear regression models predicting LVEF, average global longitudinal strain, and *E*/*A* ratio, for different metabolic biomarkers, adjusted for age (years), gender, BMI (kg/m^2^), mean SBP (mmHg), mean DBP (mmHg), diabetes, and MAFLD vs. controls, as reported in Table 4.

**Table 4:** Univariate and multivariate linear regression models predicting LVEF (%), average GLS (%), and E/A ratio for different metabolic biomarkers, adjusted for age (years), gender, BMI (kg/m2), SBP (mmHg), DBP (mmHg), diabetes, MAFLD vs. control.

Dependent variable	Predictor	B unadjusted	(95% CI)	*P*-value	B adjusted	(95% CI)	*P*-value
LVEF (%)	LPC (18:2/0:0) × 10^−6^	1.244	(0.1–2.388)	0.037	0.477	(−1.685–2.639)	0.667
	*MAFLD*	−3.152	(−6.205–−0.099)	0.047	−2.915	(−9.941–4.111)	0.42
LVEF (%)	LPC (22:6) × 10^−5^	0.45	(−1.04–1.94)	0.559	−0.47	(−2.25–1.3)	0.605
	MAFLD	−3.15	(−6.21–−0.1)	0.047	−3.99	(−10.4–2.42)	0.231
LVEF (%)	Glycyl tyrosine × 10^−6^	3.148	(2.57–3.727)	<0.001	2.367	(1.34–3.394)	< 0.001
	MAFLD	−3.152	(−6.205–−0.099)	0.047	−1.318	(−7.129–4.493)	0.658
LVEF (%)	Cer (d18:0/23:0) × 10^−4^	0.175	(−1.293–1.643)	0.816	−0.297	(−1.691–1.098)	0.679
	MAFLD	−3.152	(−6.205–−0.099)	0.047	−2.344	(−8.934–4.247)	0.49
Average GLS (%)	LPC (18:2/0:0) × 10^−6^	0.957	(0.446–1.468)	<0.001	0.154	(−0.485–0.793)	0.639
	MAFLD	−3.285	(−4.481–−2.088)	<0.001	−0.01	(−2.213–2.193)	0.993
Average GLS (%)	LPC (22:6) × 10^−5^	0.36	(−0.09–0.81)	0.126	−0.23	(−0.75–0.3)	0.403
	MAFLD	−3.28	(−4.48–−2.09)	<0.001	0.25	(−1.95–2.45)	0.828
Average GLS (%)	Glycyl tyrosine × 10^−6^	0.646	(0.148–1.143)	0.013	−0.222	(−0.65–0.206)	0.313
	MAFLD	−3.285	(−4.481–−2.088)	<0.001	0.332	(−1.583–2.247)	0.735
Average GLS (%)	Cer (d18:0/23:0) × 10^−4^	0.284	(−0.337–0.905)	0.375	−0.459	(−1.005–0.086)	0.107
	MAFLD	−3.285	(−4.481–−2.088)	<0.001	0.561	(−2.082–3.204)	0.68
E/A ratio	LPC (18:2/0:0) × 10^−6^	0.214	(0.122–0.306)	<0.001	0.133	(0.008–0.258)	0.042
	MAFLD	−0.626	(−0.839–−0.412)	<0.001	0.205	(−0.13–0.539)	0.236
E/A ratio	LPC (22:6) × 10^−5^	0.12	(0.01–0.23)	0.043	0.02	(−0.09–0.13)	0.668
	MAFLD	−0.63	(−0.84–−0.41)	<0.001	0.13	(−0.24–0.51)	0.494
E/A ratio	Glycyl tyrosine × 10^−6^	0.17	(0.092–0.247)	<0.001	0.03	(−0.066–0.125)	0.548
	MAFLD	−0.626	(−0.839–−0.412)	<0.001	−0.054	(−0.379–0.271)	0.746
E/A ratio	Cer (d18:0/23:0) × 10^−4^	0.142	(0.047–0.237)	0.005	−0.001	(−0.121–0.119)	0.988
	MAFLD	−0.626	(−0.839–−0.412)	<0.001	−0.015	(−0.447–0.416)	0.945

*BMI* Body mass index, *Cer* ceramide, *DBP* Diastolic blood pressure, *LPC* lysophosphatidylcholine, *LVEF* Left ventricular ejection fraction, *MAFLD* Metabolic-dysfunction- associated fatty liver disease, *SBP* Systolic blood pressure.

The association between glycyl tyrosine and LVEF remained statistically significant, even after adjusting for confounding factors by multivariate linear regression models 95% CI (1.34–3.394, *p*-value < 0.001). Moreover, LPC (18:2/0:0) was the only metabolic biomarker that remained statistically significant for E/A ratio after performing multivariate linear regression analysis adjusted for age, gender, BMI, SBP, DBP, diabetes, MAFLD vs. control with a 95% CI (0.008–0.258, *p*-value = 0.042). Although LPC (18:2/0:0) and glycyl tyrosine were initially associated with average GLS in univariate linear regression analysis with a *p*-value of <0.001 and 0.013, respectively, this significant association was attenuated to a non-significant value after correcting for confounding factors in multivariate linear regression analysis.

## Discussion

Although several articles including systematic review and meta-analyses discussed and evaluated cardiac systolic and subclinical systolic functions, as well as diastolic function in patients with NAFLD [23, 24], none used the newly defined criteria of MAFLD, demonstrated to identifying patients with fatty liver disease with high risk of disease progression [35]. Moreover, to the best of our knowledge, this is the first observational study to evaluate several metabolic biomarkers and their potential exerted systolic, subclinical systolic, and diastolic cardiac functions in MAFLD patients vs. controls. The present study demonstrated that MAFLD patients present with systolic, subclinical systolic, and diastolic dysfunctions. Moreover, we also demonstrated that decreased glycyl tyrosine levels are associated with left ventricular systolic dysfunction and decreased LPC (18:2/0:0) levels are correlated with diastolic dysfunction, even after adjusting for confounding factors.

Several points need to be further discussed. Firstly, we used hepatic ultrasonography along with Steatotest^TM^ (Biopredictive) to confirm hepatic steatosis, hence improving the accuracy of predicting hepatic steatosis. Studies reported that ultrasonography has a sensitivity ranging between 60-94% and specificity between 88 and 95% in detecting hepatic steatosis, possible only when hepatocyte fat accumulation is >15–20% [36, 37]. Although histopathological evaluation by liver biopsy is considered as the gold standard for identifying hepatic steatosis, it remains an invasive procedure with possible complications. Therefore, recent studies evaluated the accuracy of non-invasive biomarkers in detecting hepatic steatosis and liver fibrosis [38]. Furthermore, several articles confirmed that SteatoTest^TM^ (Biopredictive) provides a simple and non-invasive quantitative estimate of hepatic steatosis, with an AUROC of 0.811 [39, 40].

Secondly, we noticed a significant age difference between MAFLD patients and controls. This can be explained because our controls were mainly hospital staff not known to have medical illnesses. However, we corrected for this difference by including age in our multivariate linear regression models.

Thirdly, MAFLD patients were found to have a significantly increased prevalence of metabolic syndrome, diabetes, hypertension, and dyslipidemia, as well as increased BMI levels, all considered as metabolic risk factors as described by the recently defined MAFLD criteria. Obesity is known to be the primary risk factor for several non-communicable diseases, especially type 2 diabetes [41]. Accordingly, management strategies targeting both obesity and diabetes are necessary in this group of patients, achieving glycemic control, as well as weight loss. A recently published review outlined the importance of clinical care and innovative trial design for managing MAFLD and associated metabolic diseases [42]. Moreover, several adipokines such as adiponectin, leptin, and visfatin were evaluated in fatty liver disease and metabolic syndrome [43–45]. It was reported that adiponectin to leptin ratio, being known as a marker of dysfunctional adipose tissue, was significantly reduced in metabolic syndrome patients [45]. Furthermore, the leptin to adiponectin ratio and arterial stiffness were found to correlate with hepatic steatosis severity [46]. However, no significant association was reported between leptin to adiponectin ratio and arterial stiffness in NAFLD, suggesting that other pathogenic links might be related to atherosclerosis in NAFLD. An experimental study demonstrated that administering leptin can promote irisin-induced myogenesis, but suppresses the leptin-induced subcutaneous fat browning [47].

Fourthly, MAFLD patients were found to have an increased systolic and subclinical systolic dysfunction evaluated using LVEF and GLS, as well as diastolic dysfunction evaluated using several echocardiographic parameters including *E*/*e*′ and *E*/*A* ratios. These findings support the currently published articles that evaluated systolic, subclinical systolic, and diastolic cardiac functions in NAFLD patients [23, 24].

Fifthly, we performed a non-targeted metabolomic analysis, including glycyl tyrosine, LPC (18:2/0:0), LPC (22:6), and Cer (d18:0/23:0), all found to have significantly decreased levels compared to controls. Although some published articles evaluated several metabolites and metabolomic profiling in NAFLD, the current literature remains limited in data evaluating metabolic biomarkers in hepatic steatosis, and specifically with the MAFLD criteria [11, 48]. We further evaluated whether these metabolic biomarkers are associated with echocardiographic alterations. We demonstrated that LPC (18:2/0:0) is associated with *E*/*A* ratio and glycyl tyrosine is correlated with LVEF, even after adjusting for confounding factors by multiple linear regression models. Further future research is necessary in order to confirm our findings as the current literature is limited in articles evaluating these associations.

Some important potential limitations in our study should be further discussed. The cross-sectional study design does not allow us to confirm causality between the reported associations. The modest sample size did not allow us to perform subgroup analysis, limiting our evaluation of the assessed metabolic biomarkers and cardiovascular parameters in different MAFLD subtypes. As the current study was conducted in a single center, the obtained results cannot be generalized on other populations. We did not perform liver biopsy, the current gold standard, to confirm hepatic steatosis.

Nevertheless, our study has also several important strengths. We combined ultrasonography and SteatoTest^TM^ (Biopredictive) to confirm the presence of hepatic steatosis, thus improving the accuracy of predicting hepatic steatosis. Moreover, we used the newly defined criteria for MAFLD, that was demonstrated to identify fatty liver disease patients with an increased risk of disease progression [35]. We evaluated several metabolites using UHPLC-MS. To the best of our knowledge, this is the first study to evaluate cardiac systolic, subclinical systolic, and diastolic cardiac functions in MAFLD patients, as well as evaluating several metabolic biomarkers in predicting these cardiac functions. We believe that this study is of clinical significance as it demonstrates that MAFLD patients are at increased risk of developing cardiovascular disease, thus requiring further assessment in order to prevent further cardiovascular complications, and associated increased morbidity and mortality rates.

Patients with MAFLD present an increased risk for developing cardiac systolic and subclinical systolic dysfunction, as well as diastolic dysfunction. Decreased glycyl tyrosine levels were demonstrated to be associated with reduced left ventricular systolic function and decreased LPC (18:2/0:0) levels were correlated with diastolic dysfunction, even after adjusting for confounding factors.

Further observational studies with larger sample sizes are deemed necessary in order to validate and confirm the reported results. If the demonstrated findings can be further confirmed, these metabolic biomarkers can be used as potential markers to evaluate cardiac systolic and diastolic functions in MAFLD patients. Thus, playing an essential role in the detection of early cardiovascular disease in MAFLD patients, by reducing or preventing further cardiovascular complications and mortalities.
